# Sprayable tissue adhesive with biodegradation tuned for prevention of postoperative abdominal adhesions

**DOI:** 10.1002/btm2.10335

**Published:** 2022-05-23

**Authors:** Metecan Erdi, Selim Rozyyev, Manogna Balabhadrapatruni, Michele S. Saruwatari, John L. Daristotle, Omar B. Ayyub, Anthony D. Sandler, Peter Kofinas

**Affiliations:** ^1^ Department of Chemical and Biomolecular Engineering University of Maryland College Park Maryland USA; ^2^ Sheikh Zayed Institute for Pediatric Surgical Innovation, Joseph E. Robert Jr. Center for Surgical Care Children's National Medical Center Washington District of Columbia USA; ^3^ David H. Koch Institute for Integrative Cancer Research Massachusetts Institute of Technology Cambridge Massachusetts USA

**Keywords:** adhesion barrier, surface erosion, tissue adhesive, tuned biodegradation

## Abstract

Adhesions are dense, fibrous bridges that adjoin tissue surfaces due to uncontrolled inflammation following postoperative mesothelial injury. A widely used adhesion barrier material in Seprafilm often fails to prevent transverse scar tissue deposition because of its poor mechanical properties, rapid degradation profile, and difficulty in precise application. Solution blow spinning (SBS), a polymer fiber deposition technique, allows for the placement of in situ tissue‐conforming and tissue‐adherent scaffolds with exceptional mechanical properties. While biodegradable polymers such as poly(lactic‐co‐glycolic acid) (PLGA) have desirable strength, they exhibit bulk biodegradation rates and inflammatory profiles that limit their use as adhesion barriers and result in poor tissue adhesion. Here, viscoelastic poly(lactide‐co‐caprolactone) (PLCL) is used for its pertinent biodegradation mechanism. Because it degrades via surface erosion, spray deposited PLCL fibers can dissolve new connections formed by inflamed tissue, allowing them to function as an effective, durable, and easy‐to‐apply adhesion barrier. Degradation kinetics are tuned to match adhesion formation through the design of PLCL blends comprised of highly adhesive “low”‐molecular weight (LMW) constituents in a mechanically robust “high”‐molecular weight (HMW) matrix. In vitro studies demonstrate that blending LMW PLCL (30% w/v) with HMW PLCL (70% w/v) yields an anti‐fibrotic yet tissue‐adhesive polymer sealant with a 14‐day erosion rate countering adhesion formation. PLCL blends additionally exhibit improved wet tissue adhesion strength (~10 kPa) over a 14‐day period versus previously explored biodegradable polymer compositions, such as PLGA. In a mouse cecal ligation model, select PLCL blends significantly reduce abdominal adhesions severity versus no treatment and Seprafilm‐treated controls.

## 1 INTRODUCTION

Abdominal adhesions are deposits of dense, connective scar tissue that form between organ surfaces as a result of uncontrolled fibrogenesis following surgery, trauma, inflammation, infection, or tissue ischemia.^1^, ^2^ Such uncleaved fibrous bridges are frequently reported in the human peritoneum following surgical interventions resulting in broad serous tissue injury (e.g., abrasion, suturing), and are particularly common following abdominal surgeries such as laparotomy and appendicectomy.^3^, ^4^ Pathologic adhesion formation takes place due to an imbalance between the early fibrin deposition and degradation that occurs as part of healing after trauma, as well as the proximity of an injured surface to other structures.^5^, ^6^, ^7^ In normal abdominal tissue healing, the entire injured surface heals uniformly, and affected cells secrete numerous pro‐inflammatory cytokines, growth factors, and coagulants such as fibrin. Fibrous matrix deposition begins within 3 hours of tissue injury and increases until post‐injury Day 4 or 5, where it is then enzymatically degraded through fibrinolysis over the course of 1 week. In postsurgical adhesion formation, fibrin deposition outpaces fibrinolysis during the healing process and permanent connective adhesions are created between organs, with up to 93% of patients developing adhesions following operation in the abdomen or pelvis.^8^, ^9^, ^10^ Such unsuppressed proliferation of fibrous tissue frequently causes small bowel obstruction, female infertility, or chronic abdominal or pelvic pain and is implicated in up to 60%, 40%, and 80% of cases, respectively.^11^, ^12^, ^13^, ^14^

Removal of postsurgical adhesions through adhesiolysis can be attempted laparoscopically as to reduce frequency and severity in the abdominal cavity, but ultimately these procedures only have a ~70% success rate while also increasing the risk of new adhesion formation.^15^ Treatment of small bowel obstruction accounts for up to 1% of all general surgical admissions, 3% of all laparotomies, over $2 billion in hospitalization and surgical expenditures annually, as well as an approximate 900,000 days of inpatient care.^10^, ^16^, ^17^, ^18^ Because these surgical interventions to treat adhesions prove to be largely ineffective and costly, prophylactic barrier materials are needed that can prevent adhesions between organs before they form. Hydrogel‐based adhesion barriers are the most widely adopted tool in surgical settings, but are difficult to apply, poorly adhesive to the target organ, and degrade too quickly to effectively prevent adhesions.^13^

Currently available clinical products to prevent adhesion formation include Seprafilm (Genzyme)—a predried hydrogel film made of carboxymethylcellulose‐hyaluronic acid that swells once in contact with aqueous abdominal fluid—and Interceed (Johnson & Johnson), a woven cellulose mat. Both products act as solid barriers and physically prevent adhesions by separating injured mesothelial surfaces through interfacial lubrication imparted by their hydrophilic surface properties. Because they are prefabricated, such clinical products are brittle and difficult to apply, with limited flexibility when conforming to geometrically complex tissue surfaces. They also degrade rapidly in moist environments in the critical 5‐day maturation period for adhesions, exhibit impeded wound healing, and inability to seal sites of injury, the combination of which limits their use in clinical practice.^19^, ^20^, ^21^, ^22^, ^23^, ^24^ Furthermore, Seprafilm undergoes a 90% loss in tensile strength within 30 minutes due to swelling of its carboxymethylcellulose‐derived network, which renders it largely ineffective in the abdominal cavity where organs are in perpetual motion and tissue surfaces are routinely extending.^19^, ^20^ Recent biomaterials research efforts have recently focused on use of physically crosslinked hydrogels comprised of nanoparticles dispersed in a cellulose matrix.^25^, ^26^ However, they exhibit reduced flexibility and adherence to wet tissue, and also require an intricate syringe‐based deposition technique. Other investigated hydrogel systems include ones forming chemical crosslinks to tissue in situ via reactive end group chemistries, as the resultant material mimics biological tissue stiffness and thereby promotes biocompatible interfacing upon implantation.^27^, ^28^, ^29^ However, such materials frequently swell, causing undue pressure on surrounding tissue, and utilize crosslinking approaches that employ either toxic initiators or adhesive curing processes such as ultraviolet light and high temperature.^30^, ^31^ An implanted material for use as an adhesion barrier must not only be retained at the sight of application, but also maintain mechanical integrity during critical stages of fibrosis and wound healing.

To develop an adhesion barrier that is sprayable, tissue adhesive to only the target organ, degradable at the same rate as the abdominal tissue wound healing process, and does not impede wound healing, we investigated solution blow spinning (SBS) of dry, conformal polymer fibers with controllable surface erosion. Through blending of fast degrading low‐molecular weight (LMW) and slow degrading high‐molecular weight (HMW) surface eroding polymers at defined ratios, we can design sprayable fiber mats with linear biodegradation profiles tuned to a clinically relevant rate. Previous research investigations from our group have reported the biocompatibility and efficacy of SBS‐deposited polymer materials for in vivo surgical applications including antimicrobial burn wound dressings,^32^ sealants for intestinal anastomosis,^33^, ^34^, ^35^ and hemostats for traumatic bleeding.^36^ While stretchy, durable materials are desirable for high tissue adhesion, viscoelasticity, and tunable biodegradation are necessary to provide a matrix that facilitates complete wound healing in a moist environment. For example, cohesively strong poly(lactic‐co‐glycolic acid) (PLGA) not only displays a lack of wet tissue adherence unless blended with an additional adhesive component, but also induces abdominal adhesions in a clinical mouse model over a 10‐day time course.^37^ Such shortcomings are a result of a near 0% loss in polymer mass and remaining polymer providing a template for fibrous tissue growth. In contrast, separate biodegradable viscoelastic polymer blends were investigated as the primary dressing in a porcine partial‐thickness wound model, exhibiting high wet tissue adherence (>1 N/cm^2^) and complete wound healing in a pressure‐sensitive tissue adhesive (PSTA) application.^37^, ^38^

**FIGURE 1:**
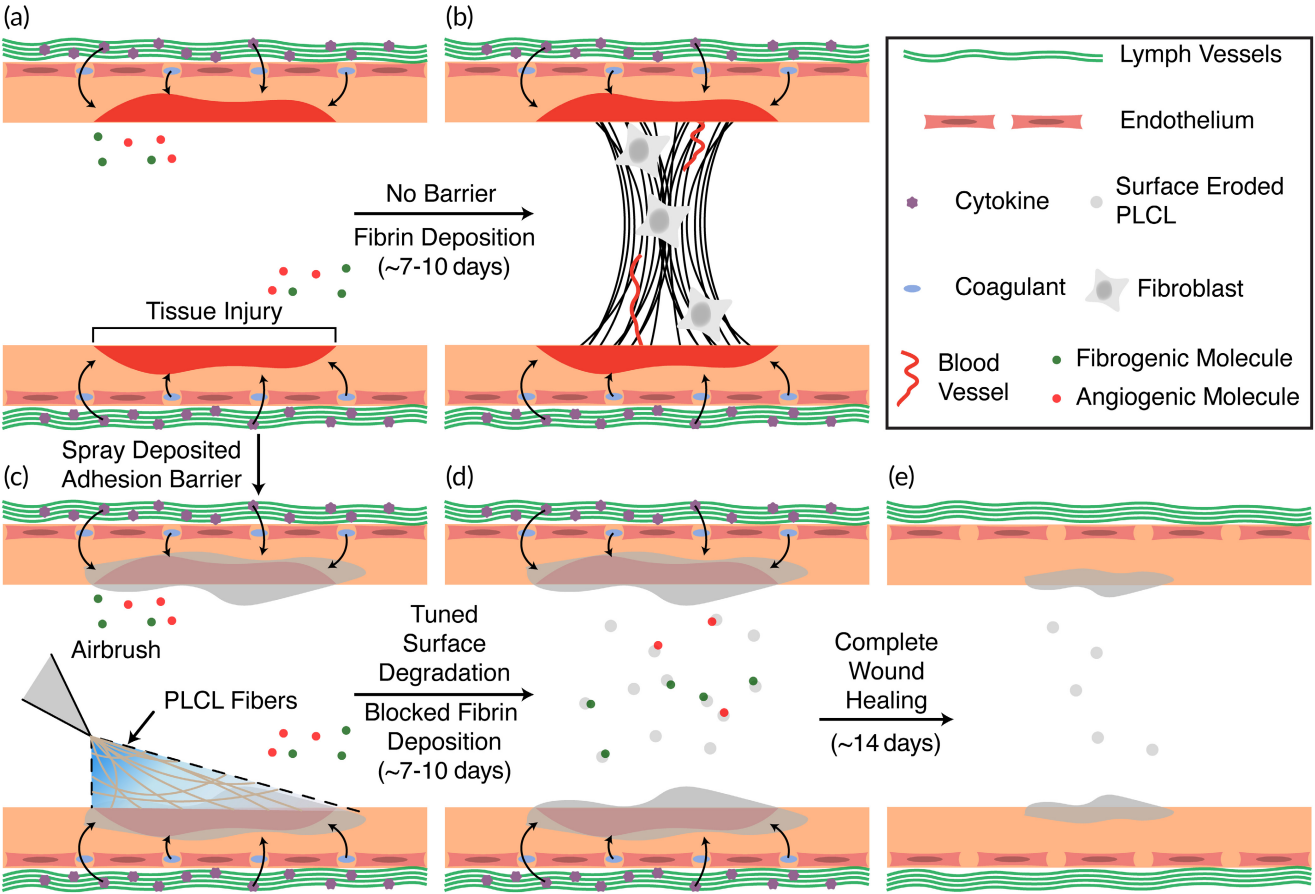
Illustration of adhesion formation in the presence of no barrier and treatment via surface eroding polymer adhesion barrier. (a) Formation of adhesions is a consequence of reduced fibrinolytic activity following ischemic mesothelial tissue injury, (b) leading to deposition of connective wound healing tissue. (c) Our poly(lactide‐co‐caprolactone) (PLCL) molecular weight blends yield a viscoelastic, wet tissue adhesive rapidly deposited via solution blowspinning (SBS) for application and retention in the abdominal cavity, while also presenting a surface erosion degradation mechanism apt to (d) prevention of adhesion formation and (e) wound healing

In this report, we studied the effect of various molecular weight blends of poly(lactide‐co‐caprolactone) (PLCL) on biodegradation profile, cohesive strength, and tissue adhesion, followed by implementation into a preclinical mouse model of abdominal adhesions. Multiple LMW and HMW combinations of PLCL were studied to modulate surface erosion rate and determine its subsequent effect on adhesion prevention. Since degraded fragments of PLCL continually erode from the surface, PLCL has the potential to act as a favorable adhesion barrier material if its degradation profile is tuned to coincide with adhesion formation (Figure 1). A constantly eroding surface will mitigate cell adhesion and fibrin deposition, which are necessary steps for the formation of adhesions.^39^, ^40^ The barrier itself—tuned to retain the necessary mechanical properties at the application site—is critical to occlude atypical deposition of fibrous, vascular scar tissue until the target wound itself has healed. We aimed to strike a balance between the presence of critical wound healing components through kinetic control of degradation, as well as necessary cohesive and adhesive strength through facile tuning of HMW and LMW ratios. PLGA was referenced as a bulk degrading control that undergoes minimal erosion during the adhesion‐forming period. First, in vitro biodegradation and mechanical testing techniques were used to determine the optimal composition of PLCL molecular weight blends for adherence to wet tissue and biocompatibility. Then, PLCL blend adhesion barriers were studied in an in vivo mouse cecal ligation model via assessment of adhesions severity and subsequent immunological analysis to demonstrate the potential benefits of a spray deposited, wet tissue‐conforming, adhesion barrier material.

## 2 RESULTS

### 2.1 Biodegradation, mechanical properties, and biocompatibility of neat and blend PLCL

Degradation of surface eroding PLCL blends was studied by immersing samples in 37°C phosphate buffered saline (PBS). Samples were then removed at select time intervals, and following a vacuum dry step, measured for mass loss, and then prepared for both molecular weight distribution analysis via gel permeation chromatography (GPC) and tensile stiffness measurements via dynamic mechanical analysis (DMA).

**FIGURE 2:**
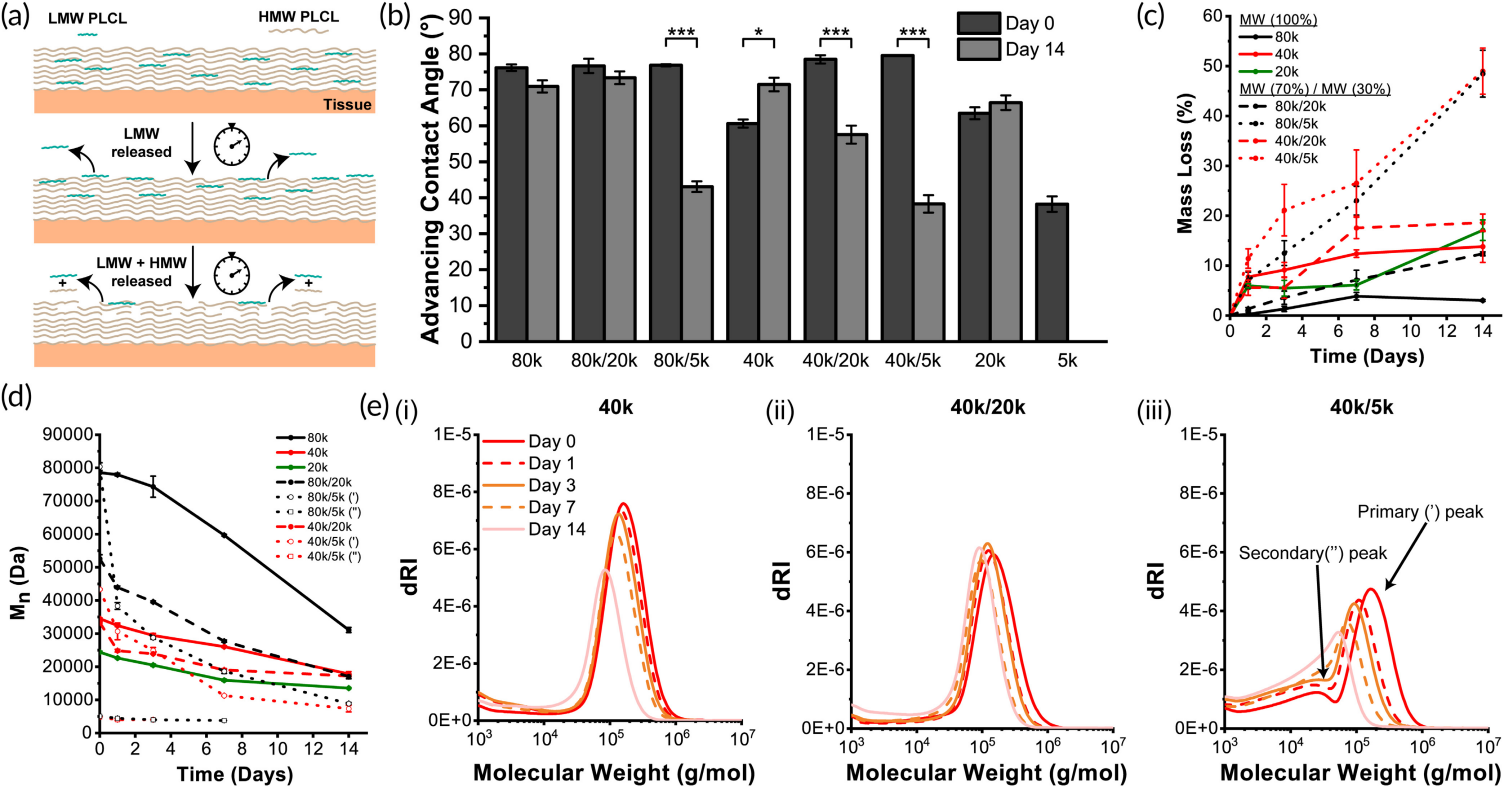
(a) Schematic of degradation mechanism for poly(lactide‐co‐caprolactone) (PLCL) via polymer surface erosion. (b) Advancing water droplet contact angle of neat and blend PLCL at start and end of in vitro degradation. (c) Mass loss for neat and blend PLCL. (d) Number average molecular weight and (e) overall distributions for PLCL blends during in vitro degradation. Blending of different molecular weights allows for tunable degradation with rapid linear degradation in the first several days for 80k/5k and 40k/5k blends. HMW = “high” molecular weight. LMW = “low” molecular weight. (′) = HMW peak of blend. (″) = LMW peak of blend. Data are plotted as mean ± SE *p < 0.05; **p < 0.01; ***p < 0.001

Molecular weight blends of surface eroding polymers present an opportunity to finely tune composite degradation profile due to the faster erosion rate of LMW chains. LMW chains in the initially fabricated fiber mat accumulate at the material surface and decrease the contact angle of blends over time (Figure 2a,b). Contact angle measurements for neat HMW PLCL are unchanged over 14 days, but they decrease significantly when blended with 5k PLCL, indicating the presentation and erosion of LMW chains with hydrophilic endgroups at the fiber mat surface.

Adhesions form within 5–7 days and then mature over 2 weeks. Any potential barrier material needs to prevent contact between surfaces during the initial stages of fibrin deposition and persist until the injured mesothelium is healed. Blending HMW 40k or 80k PLCL with LMW 5k PLCL at a 70:30 ratio results in a linear degradation profile for up to 14 days (~50% mass loss) (Figure 2b) while also displaying distinct bimodal molecular weight distributions in GPC not presented in other blends and neat compositions (Figures 2c,d, S1). As these particular blends begin to degrade and decrease in molecular weight, there is a shift to a unimodal distribution with a high PDI (~3) due to the presence of 5k PLCL as synthesized, along with degraded portions of 40k or 80k PLCL. Since all other blend compositions yield only 5%–20% mass loss and plateau in later stages, 40k/5k and 80k/5k PLCL blends have the potential to equivalently release short‐chain fragments from the polymer surface over a 14‐day treatment period for adhesions. The fast, linear erosion rate will decrease the accumulation of fibro‐ and angio‐ genic molecules, such as fibrinogen and vascular endothelial growth factor (VEGF), thereby reducing scar tissue formation on healing mesothelium.

Blending either LMW 5k or 20k PLCL in an HMW 40k or 80k PLCL matrix greatly promotes tensile elasticity as both are near (20k) or below (5k) entanglement molecular weight, while also presenting viscous behavior that permits flow upon the application of an external force. Both 40k/5k and 80k/5k PLCL blends in particular display improved adhesive strength to tissue versus their neat 40k or 80k PLCL compositions, as the 5k component allows the sealant to spread across a given surface under application of pressure. Adhesion to a surface under these conditions is facilitated through physical mechanisms of polymer chain entanglement with complex tissue topography and short‐range interactions (e.g., Van der Waals) with surface molecules as facilitated through the viscoelastic nature of our adhesive.^41^, ^42^, ^43^

**FIGURE 3:**
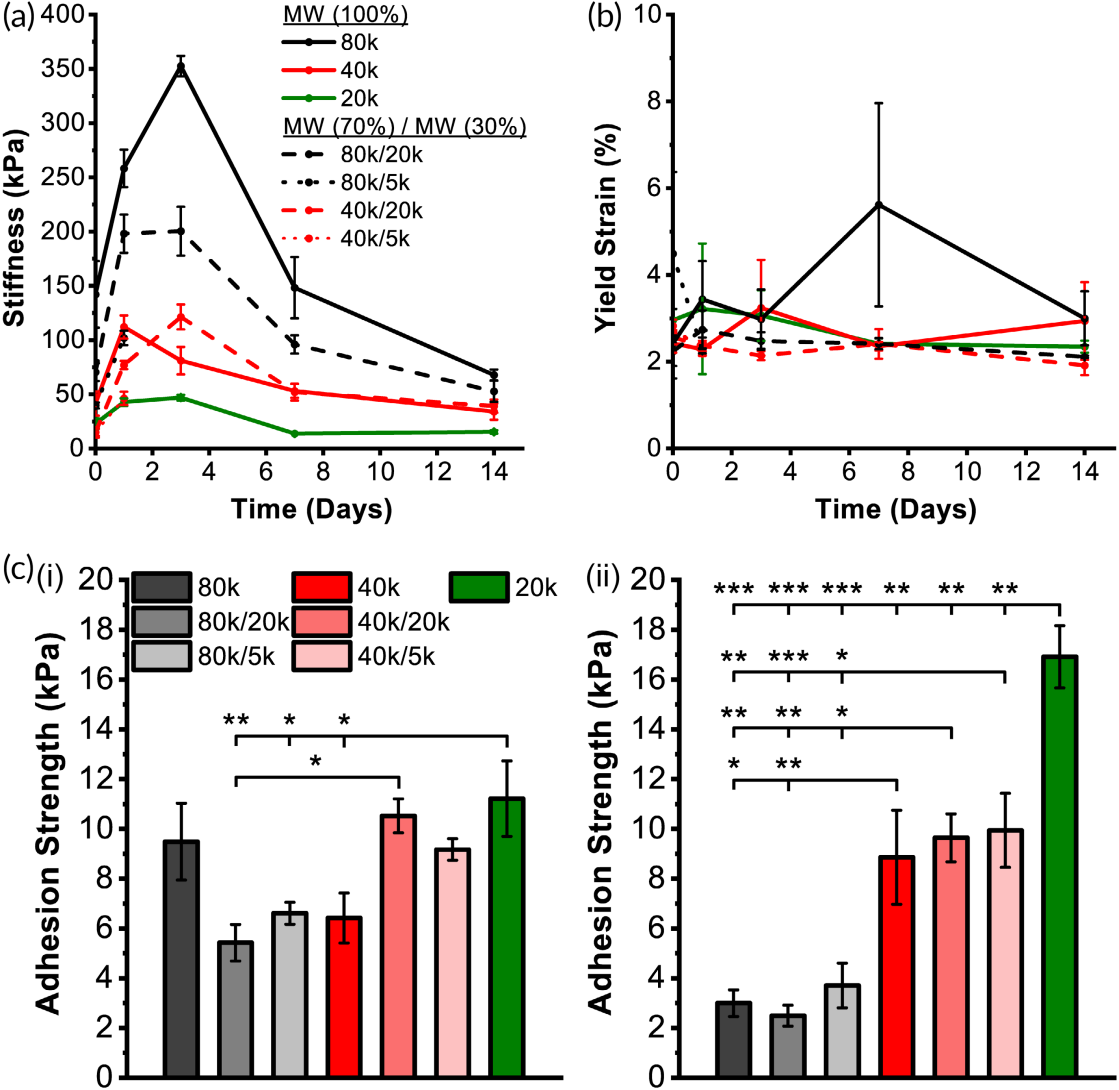
(a) Tensile stiffness, (b) yield strain, and (c) day 0 pull‐apart adhesion strength for (i) band‐aid‐to‐skin‐tissue and (ii) cardiac‐patch‐to‐intestine‐tissue of neat and blend poly(lactide‐co‐caprolactone) (PLCL) during in vitro degradation. MW, molecular weight. Both pull‐apart adhesion tests were done with 1 min of applied pressure, as to show the positive effect on tissue adherence with blending 20k or 5k PLCL. Data is plotted as mean ± SE. Asterisks indicate statistical significance: *p < 0.05; **p < 0.01; ***p < 0.001

As expected, blending 5k or 20k PLCL produces materials with decreased stiffness (Figure 3a) and values of yield stress (Figure S2) versus neat HMW compositions during in vitro degradation. Interestingly, yield strain values remain in the same order of magnitude no matter the compositions, indicating similarity in elastic range across all compositions and time points (Figure 3b). Blends of PLCL with 5k or 20k components exhibit augmented pull‐apart adhesion strength versus respective neat HMW compositions on not only dry porcine skin, but also internal wet porcine intestine (Figure 3c). Such an improvement is due to an adjusted balance between cohesive strength and adhesive strength. While neat 20k PLCL displays significantly increased adhesive strength versus other compositions on wet porcine intestine tissue, the lack of cohesive strength elucidated by tensile stiffness measurements, as well as an unfavorable nonlinear degradation profile (Figure 2c), make it a poor adhesion barrier material candidate. The equivalent biodegradation rate over 14 days (Figure 2c) for 40k/5k PLCL, coupled with superior dry and wet tissue adhesion strength (Figure 3c), makes this blend particularly promising as a favorable adhesion barrier.

### 2.2 In vivo efficacy in a mouse model of abdominal adhesions and wound healing

**FIGURE 4:**
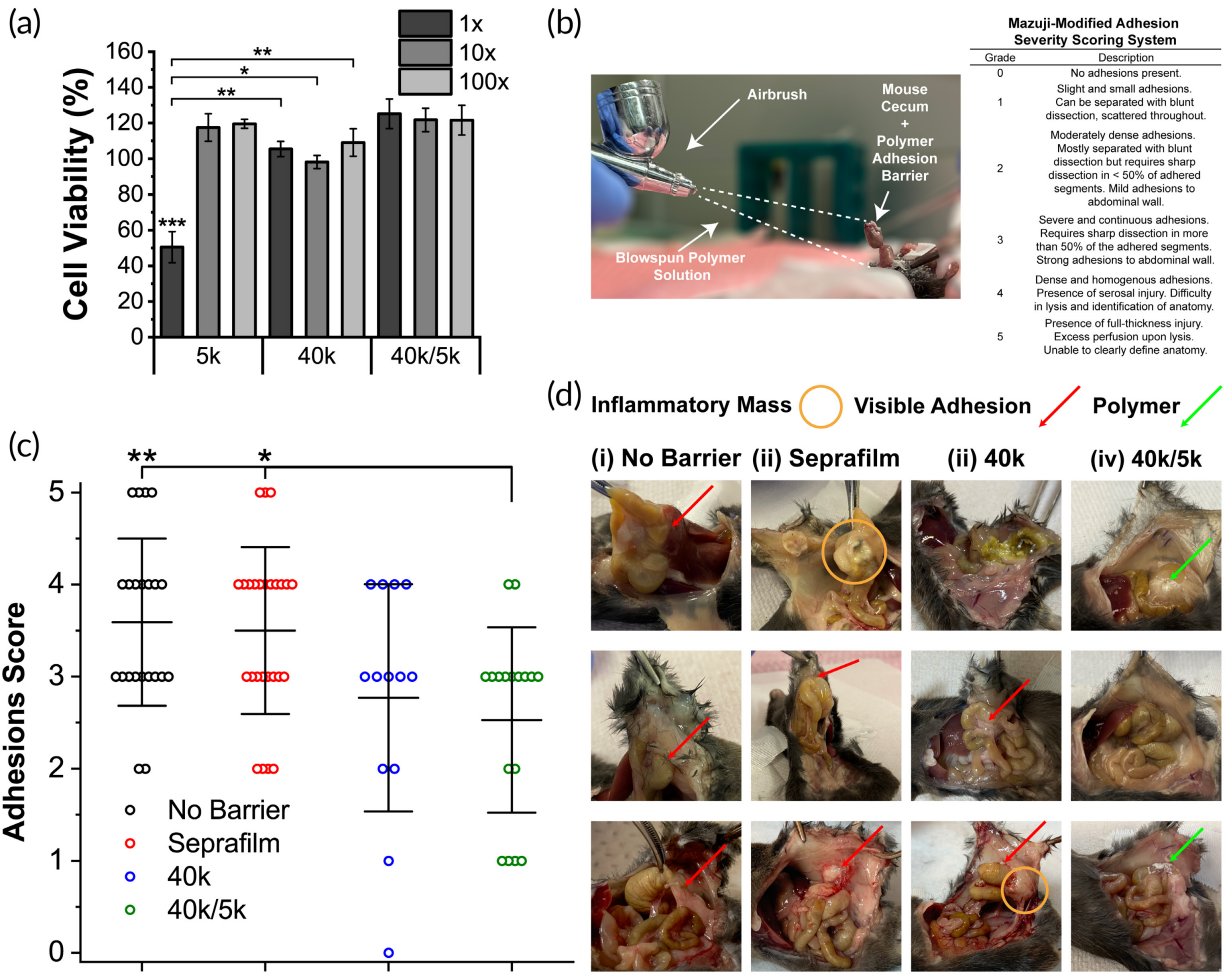
(a) L929 mouse fibroblast cell viability (vs. media only control) of neat and blended poly(lactide‐co‐caprolactone) (PLCL) for ×1, ×10, and ×100 dilutions of treatment media. (b) Application of polymer adhesion barrier during mouse cecal ligation and Mazuji‐derived adhesion scoring rubric used in clinical assessment. (c) Clinical scores and (d) gross pathology for (i) no barrier, (ii) Seprafilm, and (iii and iv) PLCL‐treated groups post‐cecal ligation at *t* = 7 days. PLCL treatment groups showed increased significance versus empty and clinical controls in reducing adhesion severity. Data are plotted as mean ± SE. Asterisks indicate statistical significance: *p < 0.05; **p < 0.01; ***p < 0.001 (5k PLCL vs. unmarked groups in cell viability)

Below entanglement molecular weight polymers (~1 kDa) formed in vivo can exhibit toxic effects due to an ability to disrupt cell membrane integrity.^44^ We therefore assessed toxicity prior to in vivo implantation of 5k PLCL in either neat or blend compositions. L929 mouse fibroblasts were treated with supernatant of degraded polymer. Neat 5k PLCL significantly reduced cell viability (~50%) of L929 mouse fibroblasts at ×1 concentration, while neat 40k and blended 40k/5k PLCL compositions had no effect on cell viability at all dilutions (Figure 4a). This indicates that 40k/5k PLCL blends have low toxicity and could be safely used as an implanted adhesion barrier material.

An accurate in vivo animal model for adhesion formation should produce consistent and reproducible mesothelial injury and ischemia. Forceful abrasion of serosal tissue lining the abdominal cavity and cecal ligation have been previously used to induce adhesions.^45^ Although more directly related to operative conditions, abrasion models are largely subjective as the amount of force applied by the operator can vary. Therefore, a cecal ligation mouse model was adopted as the procedure greatly reduces variability in the creation of local tissue ischemia via mesenteric and mesothelial disruption. After cecal ligation, mice were randomized and treated with either saline (negative control), Seprafilm (clinical control), or SBS 40k or 40k/5k PLCL polymer (treatment groups). Adhesion formation and wound healing response were assessed after 7 days. Mice that did not undergo laparotomy and cecal ligation were also assessed as a no wound control.

A surgeon, blinded to the treatment group, assessed the efficacy of SBS‐deposited fiber mats as adhesion barriers using a Mazuji‐derived scoring rubric of clinical severity (Figure 4b). 40k/5k PLCL blends significantly reduced adhesion severity versus no barrier and Seprafilm‐treated controls (Figure 4c,d), while neat 40k PLCL did not exhibit the same affect. In addition, adhesions in 40k/5k PLCL‐treated groups were more frequently described as localized and sealed off from the surrounding space in blinded assessment, with fewer involved organ systems and amassed pockets of inflammatory exudate versus the 40k PLCL treatment group (Figure S3). Such a contrast in adhesions prevention efficacy between the two polymer groups versus control groups is likely attributable to differences in biodegradation profiles (Figure 2c) and tensile strength (Figure 3a). 40k/5k PLCL balances cohesive strength and strong tissue adhesion at the site of injury with rapid erosion, which mitigates the adherence of cells and fibrin that lead to the formation of adhesions.

**FIGURE 5:**
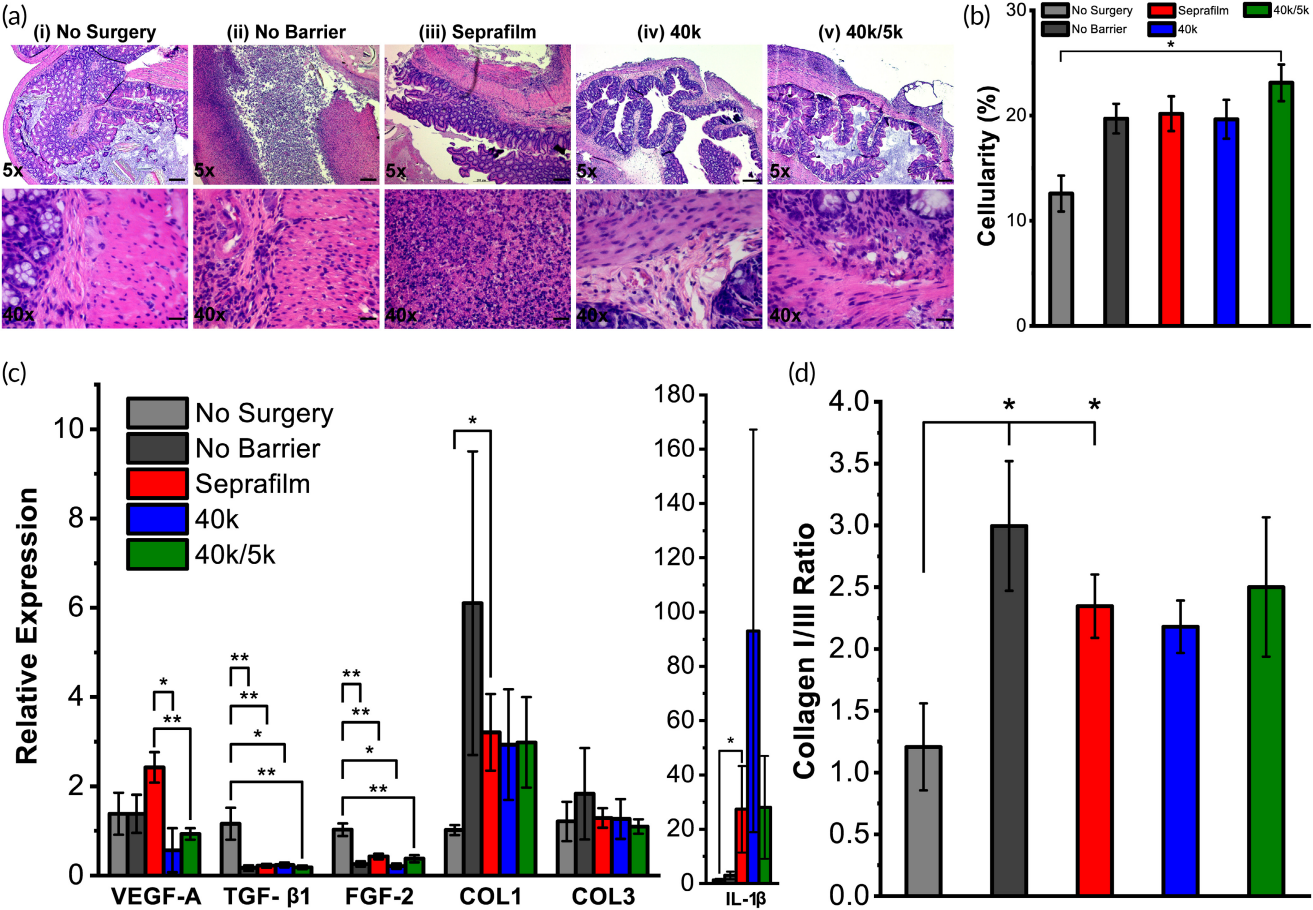
(a) Histological cross‐sections and (b) cellularity of mouse cecum, and (c) mRNA expression levels measured via RT‐PCR of vascular endothelial growth factor (VEGF‐A), transforming growth factor‐β1 (TGF‐β1), fibroblast growth factor‐2 (FGF‐2), collagen I (COL1), collagen III (COL3), and interleukin‐1β (IL‐1β) wound healing gene markers and (d) ratio of collagen I to III expression for (i) no surgery, (ii) no barrier, (iii) Seprafilm, and (iv and v) poly(lactide‐co‐caprolactone) (PLCL)‐treated groups post‐cecal ligation at *t* = 7 days (*n* = 4–5). Scale bars = 200 μm (top row) and 20 μm (bottom row). Data are plotted as mean ± SE. Asterisks indicate statistical significance: *p < 0.05; **p < 0.01; ***p < 0.001

Since fibrin deposition and remodeling is a process regulated by pro‐inflammatory signaling molecules,^46^, ^47^ cecal tissue was extracted from the mice at Day 7 for analysis of gene expression and histology. Histologic evidence of inflammation, which coincides with adhesion formation, or healing can be used to corroborate assessments of adhesion score severity. Hematoxylin and eosin (H&E) stained cecum displayed infiltration of neutrophils and eosinophils throughout the entire intestinal wall in all cecal ligation groups (Figures 5a,b, S4). Quantitative measurements of gross inflammation further assessed via cellularity analysis did not demonstrate significant differences between saline, Seprafilm, and polymer groups, although all cecal ligation groups displayed increased cellularity compared with the “no surgery” group, as expected.

Expression of critical wound healing genes in IL‐6, TNF‐α, VEGF‐A, TGF‐β1, FGF‐2, collagen I, collagen III, and IL‐1β were measured for ligated cecum samples after 7 days via real‐time PCR and compared to tissue from normal (“no surgery”) mice (Figure 5c, S5). Levels of angiogenic growth factors (VEGF‐A) were significantly reduced in the polymer groups versus Seprafilm. Fibrogenic (TGF‐β1 and FGF‐2) growth factors exhibited reduced expression in all cecal ligation groups versus the normal “no surgery” group. Expression levels of collagens I and III were decreased in the Seprafilm and polymer groups versus the no‐barrier saline group. Collagen I to III ratio is an indicator of scar‐forming collagen prevalent in cases of severe adhesions and was significantly elevated in no barrier saline and Seprafilm‐treated groups versus normal controls (Figure 5d).

## 3 DISCUSSION

An ideal adhesion barrier is one that is easily applied, biodegradable, facilitates complete wound healing, and prevents contact between injured surfaces while allowing for normal healing to occur. Here, we demonstrate that molecular weight blends of PLCL, a surface eroding polymer, permit facile in situ spray deposition of a flexible adhesion barrier while adequately adhering to wet tissue in a dynamic abdominal space. Sprayable “no touch” fiber deposition with SBS addresses practical concerns about imprecise application that surgeons encounter with currently available clinical adhesion barriers. Flexible and viscoelastic PLCL blends address further concerns about brittleness and durability. A biocompatible (Figure 4a) 40k/5k PLCL molecular weight blend yields a barrier that is not only a surface eroding material with an equivalent degradation rate over 14 days tuned exactly to fibrotic scar tissue deposition (Figure 2c), but also improved wet tissue adherence and tensile elasticity when compared with neat HMW components (Figure 3).

Advantages of solid adhesion barriers include an ability to withstand dynamic shear forces frequently present in vivo due to intestinal peristalsis or shifting of organs. Hydrogel‐based clinical barriers, such as Seprafilm, are hydrophilic and present surface properties capable of delaying a fibrotic response via reduction of tissue–tissue contact time. Seprafilm was chosen as the clinical control for in vivo studies. However, these cellulose‐derived dressings are inherently brittle prior to swelling due to their crystallinity, and lose significant adhesive and cohesive strength after swelling.^19^, ^20^ In addition, Seprafilm and other synthetic hydrogel‐based materials may impede wound healing and are especially difficult to use in abdominal surgery, resulting in limited usability in clinical settings.^21^, ^22^, ^23^, ^24^

Both neat 40k and 40k/5k blend compositions of PLCL exhibited an ability to reduce abdominal adhesions severity in a cecal ligation mouse model versus no barrier saline and Seprafilm controls, with 40k/5k in particular demonstrating statistical significance (Figure 4c,d). In addition to having less‐severe adhesive disease as denoted by our clinical scoring rubric (Figure 4b), mice treated with polymer had a decreased overall level of inflammation, as qualified through visible accumulation of inflammatory exudate by a blinded surgeon during clinical assessment. Although polymer groups were scored as having less‐severe disease when compared with the current FDA‐approved adhesion barrier Seprafilm, analysis of the gene expression of collagens I and III demonstrated equivalence between the Seprafilm and polymer groups with respect to wound healing extent, as all three groups displayed decreased levels versus no barrier saline controls (Figure 5).

Extent of fibrosis was assessed via histology and quantification of wound healing gene expression, where expression levels across controls and treatment groups were similar, with the exception of a significant decrease in angiogenic growth factor VEGF‐A for 40k/5k PLCL blend versus no barrier and Seprafilm controls (Figure 5). The significance of these findings, and implications for adhesion formation and healing, is not entirely clear. Perhaps assessment at *t* = 7 days, when the majority of initial fibrin remodeling had concluded, accounts for the absence of major differences. VEGF‐A could potentially be involved in angiogenic processes during both adhesion formation and normal tissue healing.

## 4 MATERIALS AND METHODS

### 4.1 Polymer solution preparation

Polymer solutions were prepared at a 20% (w/v) concentration in ethyl acetate for polymers characterized in vitro and in vivo. Previous research investigations have demonstrated appreciable biocompatibility and fibrous morphology of SBS fibers deposited from ethyl acetate and other solvents, as all liquid evaporates during the process and does not accumulate on the target substrate.^32^, ^33^ When used in its pure liquid form, ethyl acetate is regarded as a Class III solvent with low toxic potential by the United States Food and Drug Administration and the International Council for Harmonization of Technical Requirements for Pharmaceuticals for Human Use, hence the selection of ethyl acetate in all datasets.^32^, ^48^ Both neat polymer solutions and blends comprised of neat poly(d,l‐lactide‐co‐caprolactone) (PLCL) were investigated for LMW compositions terminologically defined as 5k PLCL (70:30 L:CL, acid endcap, Mn 1,000–5,000 Da, Akina) or 20k PLCL (70:30 L:CL, acid endcap, Mn 15,000–25,000 Da, Akina), and HMW compositions defined as 40k PLCL (70:30 L:CL, acid endcap, Mn 35,000–45,000 Da, Akina) or 80k PLCL (70:30 L:CL, acid endcap, Mn 75,000–85,000 Da, Akina). Polymer blends were mixed in a 70:30 mass ratio for a total of four blends of (1) 80k/20k, (2) 80k/5k, (3) 40k/20k, and (4) 40k/5k, where the leading component in the abbreviation is the majority (i.e., 70%) component of the blend and the secondary component is in minority (i.e., 30%). This ratio was selected as to remain in a similar material regime of published work using PLCL molecular weight blends for pressure sensitive tissue adhesive (PSTA) applications where multiple ratios were studied.^37^, ^38^ An airbrush (Master Airbrush, G222‐SET, 0.2 mm nozzle diameter) was used to deposit the solutions as dry, conformal polymer fibers. The airbrush was connected to a compressed CO_2_ tank equipped with a pressure regulator set to 20 psig.

### 4.2 Mass loss and degradation testing

Polymer samples were produced by solution blow spinning (SBS) onto a 22 mm by 22 mm glass coverslip, with the distance between airbrush nozzle and cover slip at approximately 10 cm. Polymer samples for mass loss studies were produced by spraying 2 mL of polymer solution onto a coverslip. A microbalance (Sartorius ME‐5) was used to determine the net increase in mass after the spinning process was complete, which is defined as the initial sample mass, *m*_*i*_. Samples submerged in 4 mL of ×1 PBS in wells of a 6‐well plate and stored in a shaker incubator at 37°C and 100 rpm. Samples were removed at time points of 1, 3, 7, and 14 days. At these points, the PBS was removed, and the samples were stored in a vacuum desiccator for 3 days. The samples were weighed again to determine the final mass, *m*_*f*_, and mass loss (*m*_*i*_ − *m*_*f*_) was calculated as a percentage of *m*_*i*_. Five samples were used for each time point and polymer composition (*n* = 5).

### 4.3 Gel permeation chromatography

Polymer samples from time points of degradation (1, 3, 7, and 14 days) and nondegraded samples (i.e., 0 days) were dissolved at 3 mg/mlL in tetrahydrofuran (THF). Samples were run on a Waters e2695 Separations Module with a Waters 2414 Refractive Index Detector, and Waters HSPgel columns in series (HR MB‐L and HR 3.0 columns, 6.0 mm I.D. × 15 cm). Molecular weight is reported as polystyrene relative molecular weight, as calculated from a 10‐point calibration curve generated using Agilent EasiCal polystyrene standards dissolved at 2 mg/mL in THF. GPC analysis was performed using Waters Empower 3 Chromatography Data software. The weight‐average molecular weight (Mw), number average molecular weight (Mn), and polydispersity index (PDI) of each sample were then obtained from the sample curves and recorded. Each sample type was replicated three times (*n* = 3).

### 4.4 Tensile strength testing

Tensile strength testing was performed to determine the mechanical properties of the polymer samples over time. For the 0‐day (i.e., nondegraded) experiment, samples were produced by spraying 2 mL of polymer solution onto a glass coverslip. For 1, 3, 7, and 14‐day timepoints, polymer samples were degraded according to the procedure described in the degradation testing section, removed from the coverslips, and trimmed to a rectangular shape approximately 10 mm by 5 mm in size. Exact sample dimensions were measured immediately prior to testing. Tensile testing was performed on a TA Instruments DMA Q800 equipped with a film tensile clamp. Samples were stretched under a controlled force ramp from 0 to 5 N at a rate of 0.01 N/min and measurements made at room temperature. Elastic modulus was calculated from the linear region of the resulting stress versus strain curve, with a 0.2% offset used to calculate sample yield stress and strain. Each sample type was replicated 5 times (*n* = 5).

### 4.5 Pull‐apart adhesion testing

Pull‐apart testing was performed on a TA Instruments DMA Q800. For testing on porcine skin, CVS Health Plastic One‐Size Bandages were placed in baths of ethanol to remove the adhesive. For testing on porcine intestine, Gore‐Tex Cardiovascular Patch (polytetrafluoroethylene, Gore Medical) were used as is. Both types of substrate material were cut into 8 mm square segments with 1 mL of polymer solution sprayed onto each, and 1 mL sprayed onto a section of either porcine skin or intestine. Polymer‐coated band‐aids and cardiac‐patch sections were allowed to set for 15 min in 37°C ambient air. Square sections of 8 mm frozen porcine skin or intestine were cut and warmed to room temperature by coating the tissue in water and letting the tissue warm for 10 min in 37°C ambient air. Warmed polymer‐coated substrates were brought into contact with porcine skin or intestine and superglued to the clamps of the dynamic mechanical analyzer in compression mode—the porcine skin to the fixed clamp and the polymer‐coated or uncoated band‐aid to the movable clamp. The samples were compressed at 1 N for 5 min and after this compression period a controlled force ramp was used to increase pull‐apart force at a rate of 1 N/min until failure. The adhesion strength of each sample was recorded. Each sample type was replicated five times (*n* = 5).

### 4.6 Cell viability

Cytotoxicity of polymer compositions was tested against L929 mouse fibroblasts by an elution method as described by ISO‐10993‐5.^49^ 40k/5k PLCL blend and neat 40k PLCL and 5k PLCL compositions were sprayed onto sterile 22 mm by 22 mm glass coverslips. The polymer mats were then removed from the coverslips and eluted at mass concentration of 10 mg/ml in culture media of Dulbecco's modified Eagle medium supplemented with 10% fetal bovine serum (Gemini Bio‐Products Inc.), l‐glutamine and 1% penicillin and streptomycin at standard conditions (37°C, 5% CO_2_) for 24 h. The elutions were diluted to ×1, ×10, and ×100 dilutions, and cell viability was tested against the different dilutions.

L929 fibroblasts (10^5^ cells/ml) were plated into 96‐well plates at 100 μL per well and incubated for 24 h under standard conditions. The culture media was removed by pipette. Finally, wells were then treated to control (standard media), 25 μg/mL puromycin, or diluted elutions of 40k/5k PLCL blend, neat 40k PLCL, and neat 5k PLCL compositions. This measurement was repeated five times for each diluted elution (*n* = 5).

### 4.7 Mouse cecal ligation adhesions model

All animal procedures were approved by the Children's National Hospital Institutional Animal Care and Use Committee (IACUC protocol #000030703), and the animals were treated in accordance with PHS Policy on Humane Care and Use of Laboratory Animals, the National Institute of Health Guide for the Care and Use of Laboratory Animals, and the Animal Welfare Act. Forty, 7–15‐week‐old C57BL/6 female mice were used (Jackson Laboratory). Mice were randomized into groups based on treatment group. Normal saline injection was used as a negative control, while Seprafilm® (Genzyme) was used as an antiadhesion, clinical control. Experimental endpoint was 7 days after surgery with a total of five mice (*n* = 5) allocated per group. Polymer solutions of 40k/5k PLCL blend and neat 40k PLCL were made under sterile conditions in a biosafety cabinet, and later sterilized by UV irradiation in their respective vials. Prior to surgery, a dedicated airbrush was sterilized with ethanol and placed under UV radiation along with polymer solutions.

All mice were anesthetized with a 100 mg/kg ketamine and 10 mg/kg xylazine solution (0.1 ml/10 g mouse mass). After anesthesia, the mice were positioned supine, and their skin prepped with betadine solution. In sterile fashion, a 1 cm laparotomy incision was made at the midline. After dissection into the abdominal cavity, the cecum was exposed and ligated with 4–0 Vicryl® Suture (Ethicon) approximately 1 cm from the distal end. In the case of normal saline injection, cecum was placed back into the abdominal cavity and 0.1 mL sterile saline was dripped onto ligated cecum. For the Seprafilm control group, the cecum was placed back into the abdominal cavity and a 1 cm square section gently placed on top of ligated cecum. For polymer treatment groups, 0.5 mL of solution was sprayed onto ligated cecum prior to replacement in abdominal cavity. Upon reinsertion of cecum, skin was closed using 4–0 Vicryl® Suture (Ethicon) in a running fashion, and approximately 0.1 mL buprenorphine was given as an analgesic at the end of the surgery.

Each animal was weighed both preoperatively and at 7 days after initial surgery prior to euthanasia. Midline laparotomy was performed posteuthanasia, and images of the abdominal cavity were taken with a camera. The abdominal space was then examined by a surgeon who was blinded to treatment groups and assessed for adhesions formation with scores on a Mazuji‐derived scale assigned to each attached organ pair, as well as signs of inflammation and degradation of the polymer sample.^50^

### 4.8 Histological analysis

Ligated cecum tissues were harvested on postoperative Day 7 and kept in 10% neutral buffered formalin until histological processing (Histoserv Inc.), then embedded in paraffin wax. Five‐micrometer sections were prepared, fixed onto glass slides, and stained with hematoxylin and eosin (H&E). Digital images of the histology slides were taken with TissueScope LE (Huron Digital Pathology) at ×5 and ×40 magnification then the ×40 images were exported for analysis of intestinal wall cellularity. One section per mouse, with five separate low‐ and high‐powered fields of view were imaged per section. Using ImageJ (National Institutes of Health), images were scaled to 1 μm/pixel and converted to an RGB stack. A threshold of 100 was set, and the percent area of the image stained purple was obtained for each image. These percentages were then averaged for each mouse.

### 4.9 Wound healing gene expression

RNA was extracted from frozen cecal tissue using Trizol reagent (Life Technologies, Frederick, MD). In all experiments, 6 μg RNA was used to synthesize first‐strand cDNA using High‐Capacity cDNA Reverse Transcription Kit (Life Technologies). Real‐time PCR was performed using TaqMan® Gene Expression Master Mix (Life Technologies) in a QuantStudio7 Flex RT‐PCR system (Thermo Fisher Scientific, Waltham, MA), according to the manufacturer's instructions. Reactions were performed in triplicate, including no‐template and endogenous control using GAPDH. Gene‐specific assays were Mm00434228_m1 for Il1b, Mm0046190_m1 for Il6, Mm00443258_m1 for Tnfa, Mm00437306_m1 for Vegfa, Mm01178820_m1 for Tgfb1, Mm00433287_m1 for Fgf2, Mm00801666_g1 for Col1a1, Mm00802305_g1 for Col3a1, and Mm99999915_g1 for Gapdh (Life Technologies, Thermo Fisher). Changes in relative gene expression normalized to GAPDH levels were determined using the ΔΔCt method. First, the difference between the Ct values (ΔCt) of the gene of interest and the housekeeping gene was calculated for each sample. Then the ΔCt values for the control samples were averaged. The difference in the ΔCt values between each experimental sample and the control sample (ΔΔCt) was calculated. The fold‐change in expression of the gene of interest compared to the housekeeping gene for each sample was calculated as 2^−ΔΔCt^, and the results were averaged for graphical representation.

### 4.10 Contact angle

Surface wettability was characterized by water contact angle measurements at room temperature, with images captured on a Sony a7R IV D3400 (Sony) and subsequent analysis performed in ImageJ (National Institutes of Health). Nondegraded (Day 0) and degraded polymer samples (Day 14) were prepared as described above. Advancing contact angle of 10 μl droplets of deionized (DI) water was measured using the sessile drop technique. Five samples were used for each polymer composition and time point (*n* = 5).

### 4.11 Statistical analysis

Statistical analysis was performed on Origin (OriginLab). Typically, one‐way ANOVA was used to compare group variation, followed by post hoc pairwise Tukey comparison to determine significant differences between the groups. Typically, averages were plotted with error bars representing standard error (SE). Asterisks are used to indicate statistically significant differences: *p < 0.05, **p < 0.01, ***p < 0.001. If no asterisks are shown, there are no significant differences among the groups. Real‐time PCR results were analyzed using *t*‐tests comparing the ΔΔC*t* values.

## 5 CONCLUSION

The most common adhesion barriers currently utilized in surgery are hydrogels like Seprafilm, due to their fast biodegradation and ability to interface with tissue in a biocompatible fashion. These dressings are often brittle and fracture during the application, resulting in the placement of irregular fragments that are difficult to apply and frequently fail to adhere to the intended tissue site. This work demonstrates that PLCL molecular weight blends are an effective tissue adhesive adhesion barrier material, preventing fibrosis through controlled polymer surface erosion. Spray deposition of such solid barriers via SBS allows for simple and rapid application of a conformal biomaterial with substantial cohesive and adhesive strength. The use of PLCL blends as an adhesion prevention tool is effective in reducing adhesion severity and facilitating complete wound healing as evidenced in a cecal ligation mouse model. Future studies should explore the effects of surface eroding polymer formulations on tissue healing, as these polymers have the potential to prevent leakage by sealing tissues while also inhibiting adhesion formation.

## AUTHOR CONTRIBUTIONS

**Metecan Erdi:** Conceptualization (lead); data curation (lead); formal analysis (lead); investigation (lead); methodology (lead); validation (equal); visualization (equal); writing – original draft (lead); writing – review and editing (lead). **Selim Rozyyev:** Conceptualization (supporting); data curation (equal); formal analysis (equal); investigation (equal); methodology (supporting); validation (supporting); visualization (supporting). **Manogna Balabhadrapatruni:** Formal analysis (equal); investigation (equal); methodology (supporting). **Michele S. Saruwatari:** Formal analysis (equal); investigation (equal); methodology (supporting); visualization (supporting); writing – review and editing (supporting). **John L. Daristotle:** Conceptualization (equal); visualization (equal); writing – review and editing (equal). **Omar B. Ayyub:** Conceptualization (lead); data curation (supporting); funding acquisition (equal); project administration (equal); visualization (supporting). **Anthony D. Sandler:** Conceptualization (lead); data curation (equal); funding acquisition (lead); methodology (suppoorting); project administration (equal); resources (lead); supervision (lead); validation (suppoorting); visualization (suppoorting); writing – review & editing (supporting). **Peter Kofinas:** Conceptualization (lead); data curation (equal); funding acquisition (lead); methodology (supporting); project administration (equal); resources (lead); supervision (lead); validation (supporting); visualization (supporting); writing – review and editing (supporting).

## FUNDING INFORMATION

Research reported in this publication was supported by the National Institute of Biomedical Imaging and Bioengineering of the National Institutes of Health under Award Number R01EB019963. The content is solely the responsibility of the authors and does not necessarily represent the official views of the National Institutes of Health.

## CONFLICT OF INTEREST

The authors have no conflicts of interest.

### PEER REVIEW

The peer review history for this article is available at https://publons.com/publon/10.1002/btm2.10335.

## Supporting information


**Figure S1** (a) (i) Weight average molecular weight and (ii) polydispersity index (PDI) for neat and blend poly(lactide‐co‐caprolactone) (PLCL) during *in vitro* degradation. (b, c) Overall distributions for PLCL blends during in vitro degradation. (‘) = HMW peak of blend. (”) = LMW peak of blend. Data is plotted as mean ± SE.
**Figure S2**. Yield stress values of neat and blend poly(lactide‐co‐caprolactone) (PLCL) during in vitro degradation. Data is plotted as mean ± SE.
**Figure S3.** Total number of adhesions per mouse for (i) no barrier, (ii) Seprafilm, and (iii) and (iv) poly(lactide‐co‐caprolactone) (PLCL) treated groups post‐cecal ligation at t = 7 days. Data is plotted as mean ± SE. Asterisks indicate statistical significance: *p < 0.05.
**Figure S4.** Additional histological cross sections of mouse cecum for (i) no surgery, (ii) no barrier, (iii) Seprafilm, and (iv and v) poly(lactide‐co‐caprolactone) (PLCL) treated groups post‐cecal ligation at *t* = 7 days. Scale bars = 200μm (top row) and 20μm (bottom row).
**Figure S5.** mRNA expression levels measured via RT‐PCR of interleukin‐6 (IL‐6) and tumor necrosis factor‐α (TNF‐α) for no surgery, no barrier, Seprafilm, and poly(lactide‐co‐caprolactone) (PLCL) treated groups post‐cecal ligation at t = 7 days (n = 4‐5). Data is plotted as mean ± s.e. Asterisks indicate statistical significance: *p < 0.05; **p < 0.01; ***p < 0.001.

## Data Availability

The data that support the findings of this study are available from the corresponding author upon reasonable request.
