# Supplementary material for: Sprayable tissue adhesive with biodegradation tuned for prevention of postoperative abdominal adhesions
Source: Bioeng Transl Med. 2022 May 23;8(1):e10335. doi: 10.1002/btm2.10335 (PMC9842025; doi:10.1002/btm2.10335)
Supplement: Supplementary file 1 — Figure S1 (a) (i) Weight average molecular weight and (ii) polydispersity index (PDI) for neat and blend poly(lactide‐co‐caprolactone) (PLCL) during in vitro degradation. (b, c) Overall distributions for PLCL blends during in vitro degradation. (‘) = HMW peak of blend. (”) = LMW peak of blend. Data is plotted as mean ± SE. Figure S2. Yield stress values of neat and blend poly(lactide‐co‐caprolactone) (PLCL) during in vitro degradation. Data is plotted as mean ± SE. Figure S3. Total number of adhesions per mouse for (i) no barrier, (ii) Seprafilm, and (iii) and (iv) poly(lactide‐co‐caprolactone) (PLCL) treated groups post‐cecal ligation at t = 7 days. Data is plotted as mean ± SE. Asterisks indicate statistical significance: *p < 0.05. Figure S4. Additional histological cross sections of mouse cecum for (i) no surgery, (ii) no barrier, (iii) Seprafilm, and (iv and v) poly(lactide‐co‐caprolactone) (PLCL) treated groups post‐cecal ligation at t = 7 days. Scale bars = 200μm (top row) and 20μm (bottom row). Figure S5. mRNA expression levels measured via RT‐PCR of interleukin‐6 (IL‐6) and tumor necrosis factor‐α (TNF‐α) for no surgery, no barrier, Seprafilm, and poly(lactide‐co‐caprolactone) (PLCL) treated groups post‐cecal ligation at t = 7 days (n = 4‐5). Data is plotted as mean ± s.e. Asterisks indicate statistical significance: *p < 0.05; **p < 0.01; ***p < 0.001. [file BTM2-8-e10335-s001.docx]

Supplementary Information

Sprayable Tissue Adhesive with Biodegradation Tuned for Prevention of Post-Operative Abdominal Adhesions

Metecan Erdi, Selim Rozyyev, Manogna Balabhadrapatruni, Michele S. Saruwatari, John L. Daristotle, Omar B. Ayyub, Anthony D. Sandler^*^, Peter Kofinas^*^

**Results**


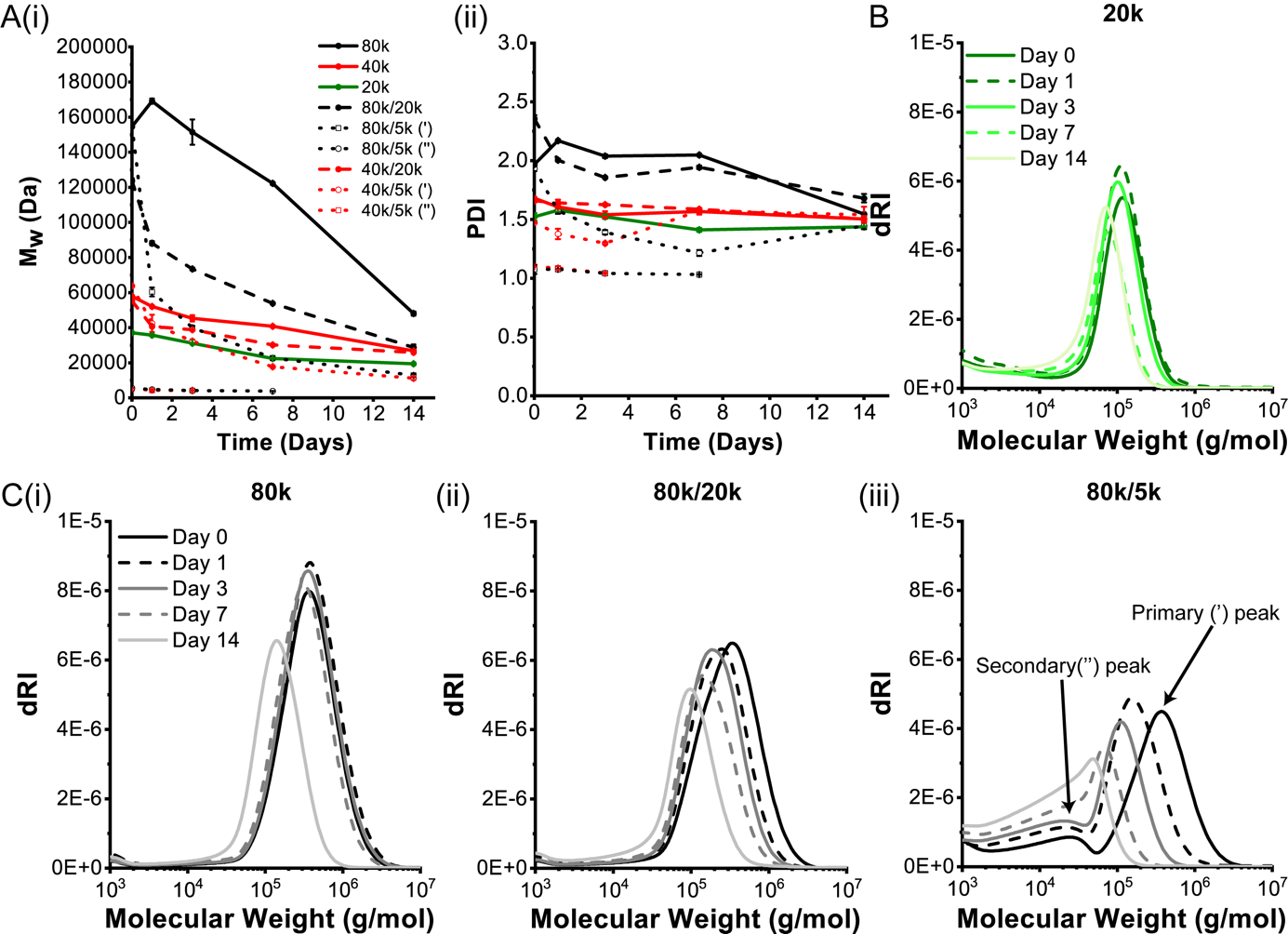


**Figure S1.** (a) (i) Weight average molecular weight and (ii) polydispersity index (PDI) for neat and blend poly(L-lactide-co-caprolactone) (PLCL) during *in vitro* degradation. (b-c) Overall distributions for PLCL blends during *in vitro* degradation. (‘) = HMW peak of blend. (’’) = LMW peak of blend. Data is plotted as mean ± s.e.


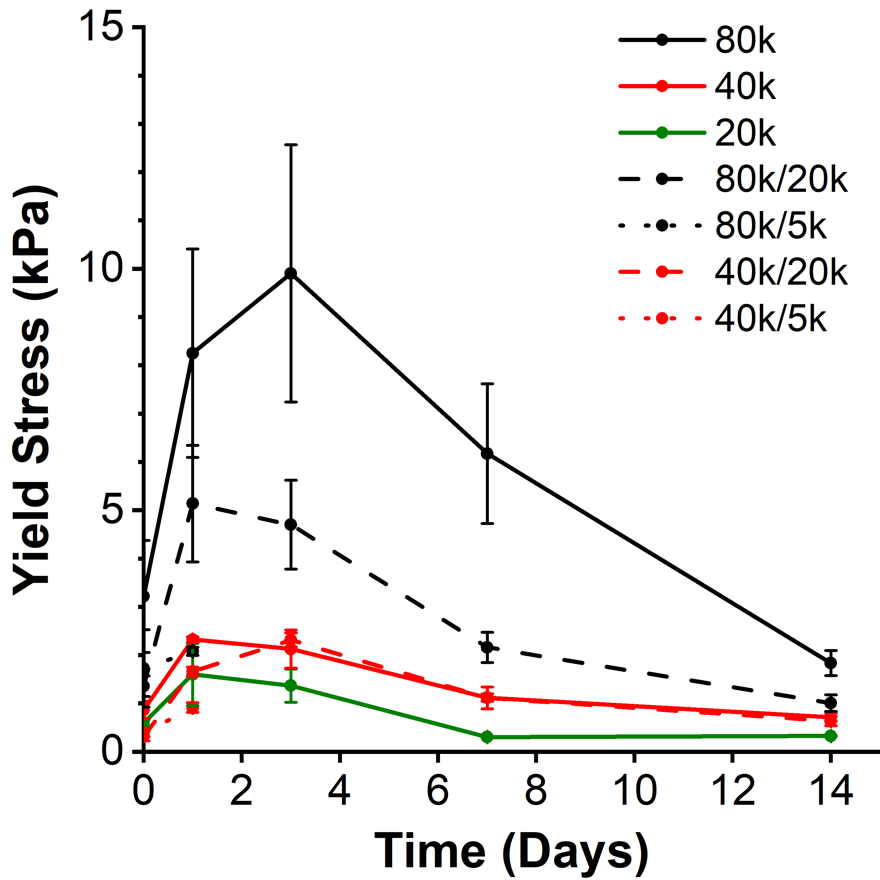


**Figure S2.** Yield stress values of neat and blend poly(L-lactide-co-caprolactone) (PLCL) during *in vitro* degradation. Data is plotted as mean ± s.e.


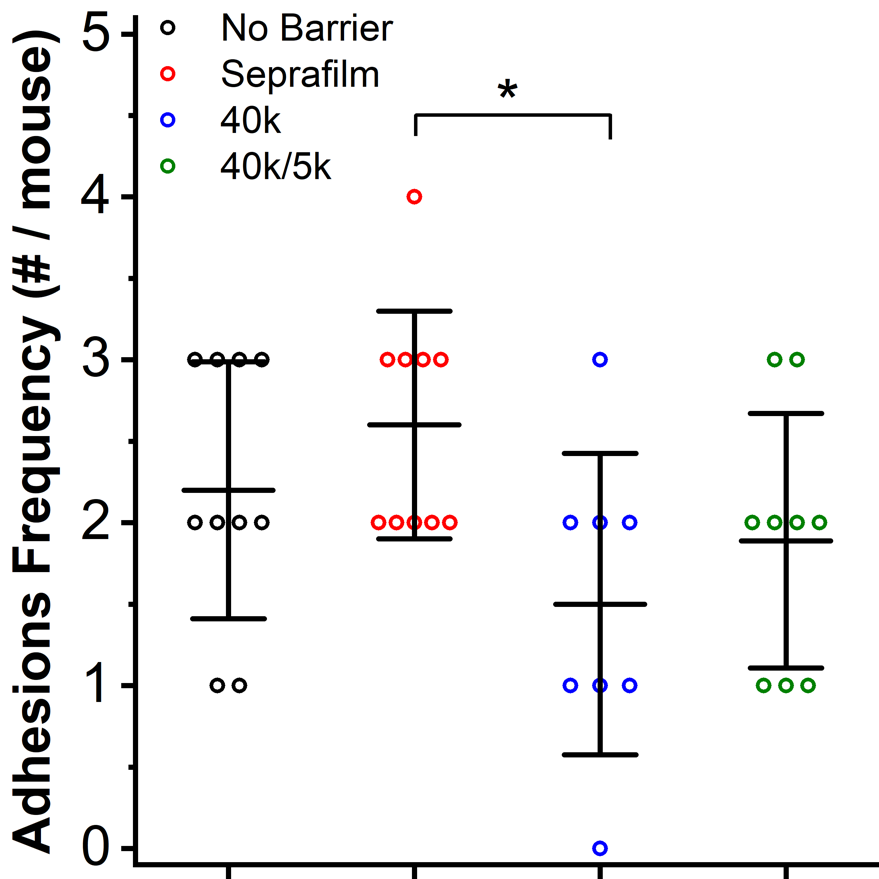


**Figure S3.** Total number of adhesions per mouse for (i) no barrier, (ii) Seprafilm, and (iii) and iv) poly(L-lactide-co-caprolactone) (PLCL) treated groups post-cecal ligation at t = 7 days. Data is plotted as mean ± s.e. Asterisks indicate statistical significance: * p < 0.05.


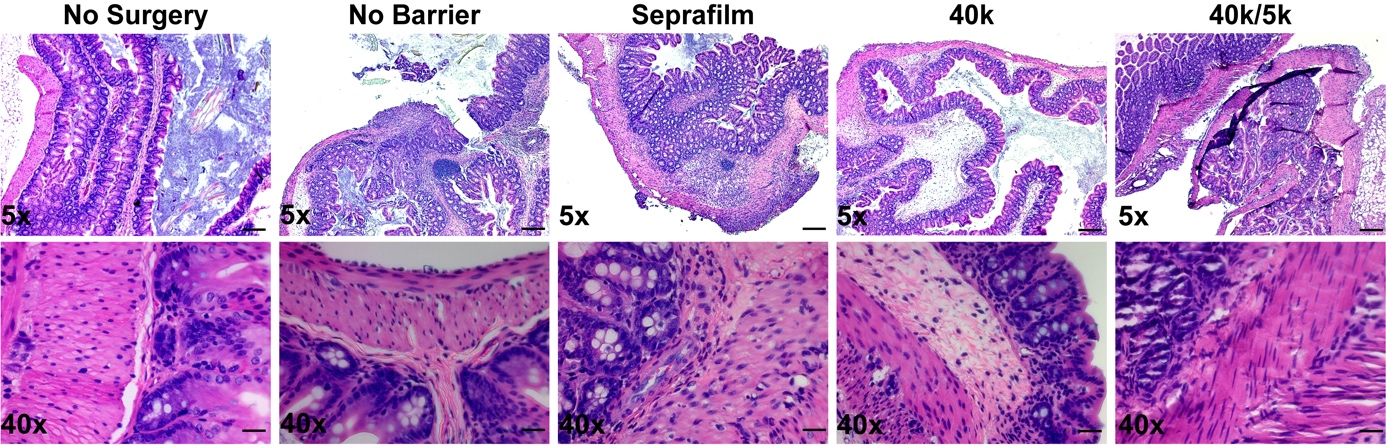


**Figure S4.** Additional histological cross sections of mouse cecum for (i) no surgery, (ii) no barrier, (iii) Seprafilm, and (iv and v) poly(L-lactide-co-caprolactone) (PLCL) treated groups post-cecal ligation at t = 7 days. Scale bars = 200µm (top row) and 20µm (bottom row).


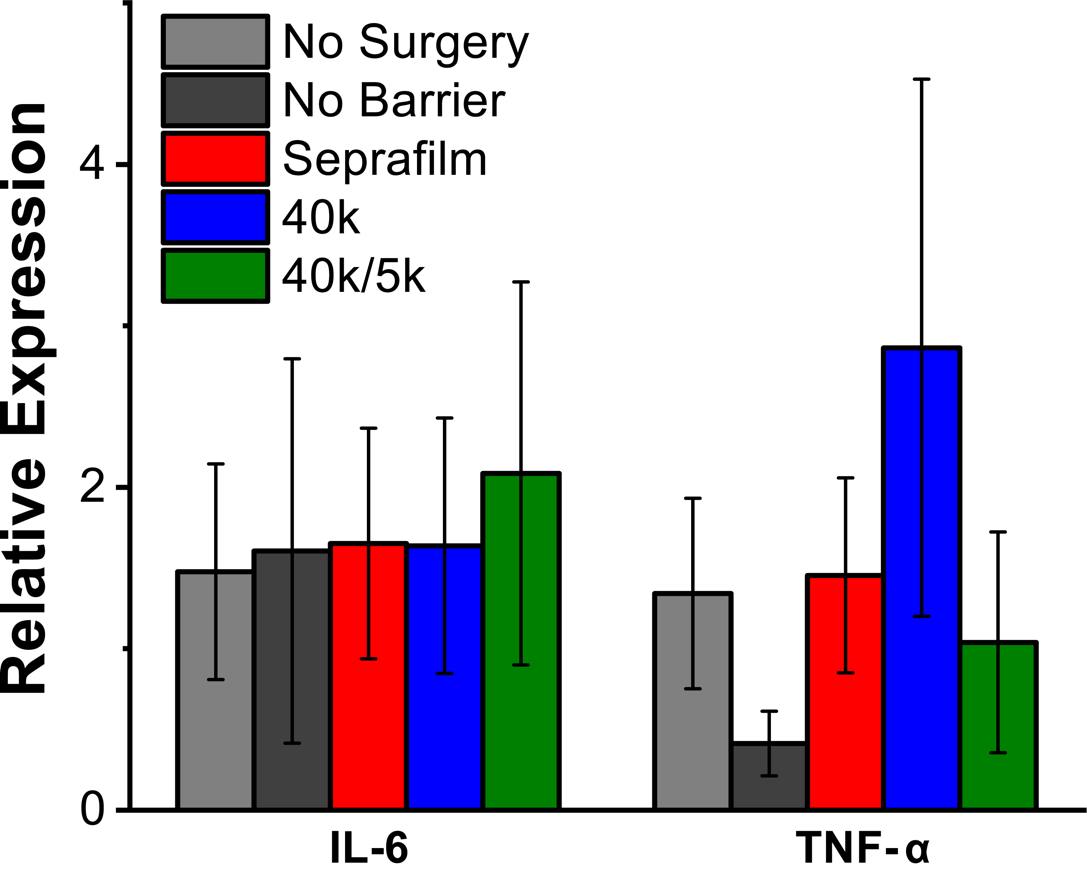


**Figure S5.** mRNA expression levels measured via RT-PCR of interleukin-6 (IL-6) and tumor necrosis factor-α (TNF-α) for no surgery, no barrier, Seprafilm, and poly(L-lactide-co-caprolactone) (PLCL) treated groups post-cecal ligation at t = 7 days (n = 4-5). Data is plotted as mean ± s.e. Asterisks indicate statistical significance: * p < 0.05; **p < 0.01; ***p < 0.001.
